# An Overview of the Adverse Impacts of Old Combs on Honeybee Colonies and Recommended Beekeeping Management Strategies

**DOI:** 10.3390/insects16040351

**Published:** 2025-03-27

**Authors:** Qingxin Meng, Rong Huang, Shunhua Yang, Wutao Jiang, Yakai Tian, Kun Dong

**Affiliations:** Yunnan Provincial Engineering and Research Center for Sustainable Utilization of Honeybee Resources, Eastern Bee Research Institute, College of Animal Science and Technology, Yunnan Agricultural University, Kunming 650201, China

**Keywords:** old comb, worker development, colony strength, immune detoxification, beekeeping management

## Abstract

The honeybee comb serves as the primary site for all essential colony activities, and its structural and functional integrity plays a crucial role in colony development. As combs age through successive brood-rearing cycles, their physicochemical properties undergo significant changes, which can negatively affect colony health and productivity. This review synthesizes the current knowledge on the biological functions of combs, the aging process, and the negative impacts of old combs on cell structure, worker morphology, colony strength, and bee product quality. Additionally, it examines the adaptive strategies employed by honeybees and the recommended beekeeping management practices to mitigate these effects. Specifically, old combs undergo structural changes in cell dimensions and reduced spatial capacity, leading to the growth of smaller bees with diminished foraging and productivity. Furthermore, bee products, such as honey and beeswax, collected from old combs demonstrate compromised quality and higher levels of environmental contaminants. To counteract these challenges, colonies engage in hygienic behaviors, such as cell cleaning and comb rebuilding, while their enhanced immune and detoxification systems help mitigate comb-derived stressors. This review demonstrates that the systematic replacement of old brood combs is a critical management strategy to optimize bee health and ensure sustainable apiculture.

## Simple Summary

Honeycombs, where bees raise their offspring and store food, naturally age over time. Old combs become darker, accumulate harmful substances like pesticides and heavy metals, and undergo structural changes that result in the shrinkage of the cells where bees develop. These changes lead to smaller workers with shorter lifespans and reduced abilities to forage, thereby weakening the entire colony’s performance. Additionally, the physical and chemical properties of honey and beeswax change as the honeycombs age. To mitigate the negative impacts of old combs, bees engage in behaviors to clean or rebuild combs, and at the body level, systemic molecular mechanisms of immune system adaptation and detoxification take place. This review highlights the importance of regularly replacing old brood combs in beekeeping practices to maintain healthy colonies, improve honey production, and reduce environmental pollution risks. By managing comb age, beekeepers can support stronger colonies and contribute to sustainable agriculture and ecosystem conservation.

## 1. Introduction

Honeybee colonies represent a cornerstone of global ecosystems and agricultural productivity, serving as indispensable pollinators for both wild flora and cultivated crops [1]. At the heart of colony functionality lies the comb—a dynamic structure meticulously engineered by bees to support brood rearing, food storage, and social communication [2]. Constructed from beeswax secreted by workers, combs undergo progressive physicochemical alterations through repeated brood-rearing cycles or from stored honey and pollen [3,4], yet the implications of these aging processes on colony health remain underexplored. While extensive research has elucidated the comb’s role in thermoregulation and nutrient storage [5,6], critical gaps persist in understanding how structural degradation and pollutant accumulation in old combs compromise individual bee development, colony resilience, and apicultural output.

Recent studies highlight alarming trends: global bee populations face unprecedented declines due to anthropogenic stressors, including habitat loss, pesticide exposure, and climate change [7,8]. Within this context, the comb emerges not only as a habitat but also as a reservoir for environmental contaminants, with old combs accumulating heavy metals, pesticides, and microbial pathogens [9,10]. These contaminants synergize with structural decay—such as reduced cell volume and thickened walls—to constrain larval development, diminish worker size, and impair foraging efficiency [11,12]. Furthermore, degraded combs compromise the quality of hive products, raising concerns for food safety and economic viability [13,14]. Despite these challenges, bees exhibit remarkable adaptive behaviors, including comb rebuilding and detoxification mechanisms, which remain poorly integrated into current apicultural practices [8,15].

This review synthesizes multidisciplinary advances to address three pivotal questions: (1) How do old combs structurally and chemically change, and what are the cascading effects on bee ontogeny and colony dynamics? (2) What adaptive strategies do bees employ to mitigate comb-derived stressors, and how can these inform sustainable management? (3) What innovations in comb renewal protocols and pollutant monitoring are critical for safeguarding bee health? By integrating empirical and experimental data from ecological and apicultural studies, this work elucidates the interplay between comb aging, colony fitness, and environmental pressures. It further proposes evidence-based strategies for comb management, emphasizing timely replacement and pollutant mitigation to enhance apicultural resilience. As pollinator declines threaten global food security, this synthesis underscores the urgency of prioritizing comb health as a linchpin for sustainable beekeeping and ecosystem conservation.

## 2. Biological Functions of Combs

The honeybee comb is a multifunctional biosynthetic architecture central to colony survival, serving as the structural and physiological cornerstone for brood development and social coordination [2]. Combs are constructed from beeswax, a complex lipids and hydrocarbons mixture secreted by the wax glands of workers. These glands, located on the ventral abdomen, convert carbohydrates from honey into wax scales through a series of enzymatic processes [16]. A honeybee nest (*Apis* spp.) is composed of one or more vertically hanging combs, spaced apart and arranged in parallel, which are crucial for maintaining the stability of the internal environment of the colony [17]. Within the dark confines of the hive, bees use the “dance language” to communicate with their peers on the comb surface about the distance and location of food resources [18].

The fundamental structural unit of the comb is the cell, which is classified into queen, worker, and drone cells based on size and shape [2]. Queen cells, specialized for queen rearing, are cup-shaped structures typically located along the lower edges of the comb, with their openings facing downward and perpendicular to the ground [19]. Worker and drone cells, primarily used for rearing workers and drones, respectively, are hexagonal prisms with bases composed of three rhombic plates [20]. The majority of the cells in the comb are worker cells, whose primary function is to provide a stable and suitable environment for the growth and development of workers. Additionally, these cells located at the upper edges of the comb can also be used to store honey and pollen, serving as a nutrient supplement for bees [21].

Furthermore, the comb and its byproducts hold significant value for human use. Beeswax is rich in bioactive compounds with therapeutic properties, including anti-inflammatory effects beneficial for conditions, such as rhinitis and hepatitis [22]. Additionally, it exhibits antioxidant, antibacterial, analgesic, and immune-enhancing activities [23]. Beyond its medicinal applications, beeswax is used as a raw material in different industries, such as coatings for food and pharmaceuticals, and cosmetics, etc. [24].

## 3. Aging Process of the Comb

**Figure 1:**
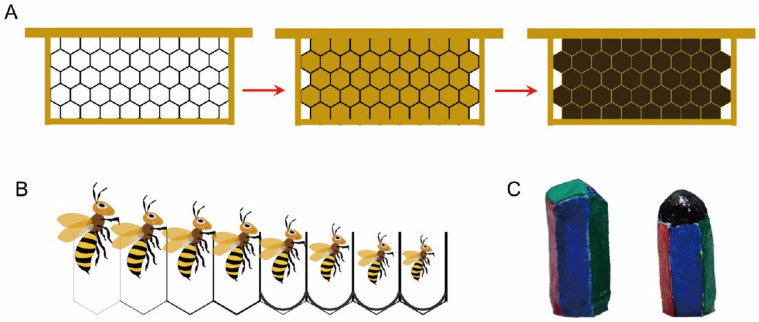
With the increase in the number of brood-rearing generations, (**A**) the comb color gradually darkens to black, (**B**) the cell volume decreases and the size of the reared worker reduces, and (**C**) the cell structure undergoes changes.

The aging of the comb is a gradual process, involving changes in both physical properties and biological functions. One of the most apparent phenotypic changes in old combs is the darkening of their color, which appears as deep brown or black (Figure 1A) [25]. Additionally, old combs influence cell structure, the morphological size of the workers reared within them, and overall colony productivity (Figure 1B,C) [3]. Old combs also tend to accumulate various harmful substances, including fungi, bacteria, pesticides, and heavy metals, which can negatively affect individual bee health and colony development [9,10].

### 3.1. Color Changes in the Comb

Beeswax, the primary component of the comb, readily adsorbs pigments from accumulated residues within the cells. Its porous structure provides numerous adsorption sites, allowing pigment particles to penetrate and adhere to the surface or interior of the wax [26]. In addition, the main constituents of beeswax include fatty acid esters (~67%), hydrocarbons (~14%), and free fatty acids (~13%) [24]. These compounds are rich in polar groups that can interact with pigments through hydrogen bonding or van der Waals forces, resulting in strong adsorption [27,28]. As combs store nectar and pollen, they gradually darken in color from their initial white or light yellow to deeper shades. This color change is also influenced by physiological activities occurring within the cells, such as fecal excretion, silk spinning for cocoon formation, and molting during bee development [29]. In particular, bee feces contain dark pigments that are easily absorbed by beeswax [11,30]. With successive brood-rearing generations, the accumulation of cocoons and other debris causes old combs to turn brown or black [25].

### 3.2. Structural Changes in the Comb Cells

Cells are the fundamental units of the comb, serving as primary sites for food storage, brood development, and adult bee rest, all of which are critical for colony development [31]. Newly constructed comb cells are typically hexagonal prisms with a base formed by three rhombic plates [32]. This design provides structural advantages, including lightweight construction, high load-bearing capacity, and space efficiency [2,17]. For instance, 1 g of beeswax can produce approximately 20 cm^2^ of double-sided comb surface, while only 55 g of wax is required to build a comb capable of supporting 1 kg of ripened and capped honey [33]. However, brood rearing leads to structural changes in the cells due to the accumulation of metabolic byproducts. With each successive generation of workers reared in a cell, residues, such as feces and cocoon silk, accumulate as they cannot be fully removed by nurse bees [11]. This accumulation increases comb weight and thickens the cell walls. Notably, the dimensions of comb cell structures vary among different bee species, leading to species-specific fluctuations in the observed changes (Table 1). For instance, the weight of a newly constructed comb of *A. m. carnica* (housed in Kafr El Sheikh, Egypt) is 0.26 g/cm^2^, but as the comb ages from 1 to 7 years, its weight gradually increases to 0.33, 0.60, 0.88, 0.95, 1.04, 1.17, and 1.32 g/cm^2^, respectively [34]. Similarly, the thickness of newly constructed cell walls in the upper part is 88 μm, but after 5 months, 1 year, and 2 years of brood rearing, it increases to 120 μm, 246 μm, and 297 μm, respectively [12].

The increase in comb weight and cell wall thickness also alters the internal shape and structural dimensions of the cells. Over time, the hexagonal prism shape of the cells transforms into a cylindrical form with a hemispherical base [32]. Compared with newly constructed brood cells, old brood cells have reduced diameter and volume [34]. For *A. m. carnica* (housed in Kafr El Sheikh, Egypt), the diameter and volume of newly constructed worker cells were measured as 6.00 mm and 0.31 mL, respectively. After 6 years of use, these dimensions decreased to 4.86 mm and 0.18 mL. A similar trend was observed in *A. c. cerana* [25]. The primary factor driving these changes is the accumulation of cocoon residue within the cells. For example, in old brood cells of *A. c. cerana* (housed in Kunming, China), the weight of the cocoon is 57.90 mg, and the thickness at the base is 0.96 mm, occupying approximately 13% of the cell space and significantly reducing the base dimensions [11]. Therefore, as the number of worker generations reared in the comb increases, the accumulation of the cocoon increases the comb weight and reduces the cell dimensions, ultimately restricting the developmental space available for bees within the cells.

### 3.3. Effects of Old Combs on the External Morphological Traits of the Workers

The structural dimensions of the brood cells are significantly correlated with the morphological characteristics of honeybees [41,42]. Old brood cells are smaller in volume compared with newly constructed cells, resulting in reduced nutrient provisioning [43,44]. Within the fixed developmental period, bee larvae reared in old cells are unable to acquire sufficient nutrients for full maturation [45]. The smaller dimensions of the old cells impose spatial stress during worker development [11]. For instance, *A. m. carnica* (housed in Somogy, Hungary) pupae that were reared in new cells exhibited significantly larger head (1.41 vs. 1.16 mm), thorax (4.74 vs. 3.60 mm), and abdomen dimensions (5.03 vs. 4.15 mm) and higher birth weights (114.89 vs. 88.05 mg) compared with those reared in old brood cells [4,46]. The birth weight is an important indicator of bee development, reflecting the size of the bee’s external morphological structure. The external morphological dimensions of bees are negatively correlated with the age of the comb in which they develop [25]. In *A. m. carnica* (housed in Hofuf, Saudi Arabia), the proboscis length of the workers reared in combs aged 1–4 years was 6.10, 5.94, 5.55, and 5.30 mm, respectively; the forewing length was 9.05, 9.03, 9.00, and 9.00 mm; the length of the third tergite was 2.30, 2.25, 2.10, and 2.05 mm; the tarsus length was 2.50, 2.48, 2.45, and 2.45 mm; and the wax mirror length was 1.55, 1.55, 1.51, and 1.50 mm [47].

The structural dimensions of the cells can influence the external morphological dimensions of bees, and vice versa. Li reported that small-sized bees reared in old combs constructed new cells with significantly reduced diameters and depths compared with large-sized bees reared in new combs [42]. Within the colony, each wax-producing bee contributes to beeswax secretion and processing, using its antennae to identify the location and quantity of wax deposition [2]. During cell construction, bees typically use their legs and antennae as measuring tools. Consequently, small-sized workers construct smaller cells due to their reduced morphological structures [48]. Additionally, the external morphology of bees is closely related to their foraging and production capabilities. Thus, the reduction in individual bee size observed in colonies using old combs may directly impair both individual foraging capacity and overall colony productivity [4,49]. However, further experimental validation is required to confirm these causal relationships.

### 3.4. Impact of Old Combs on Colony Strength and Production Performance

**Figure 2:**
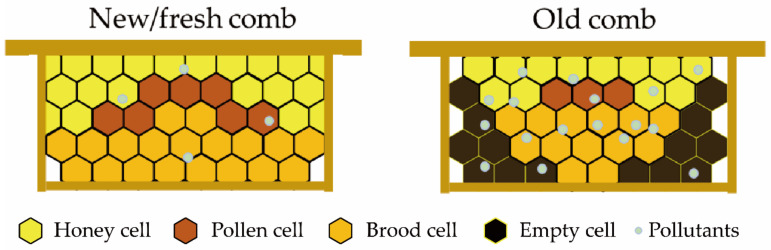
Compared with new/fresh combs, old combs exhibit a decrease in brood, nectar, and pollen, while showing an increased accumulation of pollutants.

Old combs negatively impact colony strength and production performance. Colony strength, defined as the number of workers within a colony, is a key indicator of reproductive and productive capacity [50]. Old comb significantly reduces the number of newly emerged workers and shortens their lifespan. In *A. m. carnica* (housed in Damietta, Egypt), the area of sealed broods in combs aged 1–4 years was 3722, 3698, 2985, and 2413 cm^2^, respectively; the survival rate of the sealed brood was 74.45, 71.05, 72.60, and 72.50%; and the average lifespan of the newly emerged workers was 30.0, 29.7, 27.4, and 24.0 d [3]. Therefore, colonies with predominantly new combs exhibit significantly better colony strength than those with old combs. A robust colony ensures better internal organization, with each bee performing its role effectively, leading to improved production performance [3]. The foraging frequency of workers is one of the primary factors influencing colony production performance. For example, *A. m. carnica* (housed in Al-Ahsa oasis, Saudi Arabia) colonies with 1-year-old combs had an average of 19.85 workers returning with pollen per minute, which was significantly higher than that of colonies with 2-year-old combs, which was 16.95 [51]. This may be due to the larger size and better developmental condition of workers reared in new combs, resulting in differences in flight speed and foraging efficiency. Similarly, colonies established with new or old combs exhibit differences in food storage capacity. *Apis m. carnica* (housed in Kafr El Sheikh, Egypt) colonies consisting of new combs stored 159.89 and 266.55 cm^2^ of pollen and honey, respectively, whereas those with old combs stored 95.55 and 141.16 cm^2^, respectively (Figure 2) [4].

### 3.5. Impact of Old Combs on the Quality of Bee Products

The honey quality changes with the age of the comb. A study conducted at Kafr El-Sheikh University on *A. m. carnica* revealed that honey stored in 4-year-old combs exhibited increased specific gravity and viscosity compared to fresher combs [14]. Furthermore, the chemical composition of honey shifted with comb age, with reducing sugar content rising and glucose levels declining. Organoleptic evaluation, a direct method for measuring, analyzing, and interpreting food quality, revealed that honey stored in old combs is less acceptable in terms of color, odor, taste, viscosity, and sourness. The hydroxymethylfurfural (HMF) content is an important indicator of honey quality and is used to assess its freshness [52]. The HMF content in honey produced in old combs (28.8 mg/kg) was found to be significantly higher than that in honey produced in new combs (18.4 mg/kg), potentially affecting honey quality [53]. Additionally, combs can be a reservoir for various metal elements, and concentrations of K, Na, Ca, Mg, Fe, Zn, Pb, and Mn in honey collected from different combs significantly increase with comb age [54]. Furthermore, beeswax undergoes changes in quality with comb age. Old combs have fewer long-chain esters and more short-chain compounds, with the content of alkenes, dienes, and branched alkanes being significantly higher than in new combs, while that of alkanes decreases. Due to the higher volatility of alkanes compared with alkenes, alkanes gradually volatilize as the comb age increases, representing a characteristic change in the beeswax over time [55].

### 3.6. Accumulation of Environmental Pollutants in Old Combs

Bees play a vital role in terrestrial ecosystems as primary pollinators for most crops and wild plants, making them essential for maintaining wild plant communities and agricultural productivity [56]. Bee pollination significantly enhances the fruit set rate, seed set rate, yield, and quality of blueberries [57]. In 2009, bee pollination contributed an estimated $11.68 billion to agriculture in the United States [1]. However, bee populations have been declining due to global climate change, habitat fragmentation, and harmful substances released from industrial and agricultural activities, such as pesticides [8]. Foraging bees may collect food contaminated with heavy metals, pesticides, and other harmful substances, while airborne particulate pollutants can adhere to their body hairs [58]. These contaminants readily integrate with beeswax, accumulating over time and increasing in concentration as the comb ages [59]. Consequently, old combs accumulate higher levels of environmental pollutants (Figure 2). The concentrations of Cd, Cr, Ni, Pb, and Mn in combs aged 5 years increased by 56%, 63%, 65%, 78%, and 82%, respectively, compared with 1-year-old combs [9]. Additionally, the cocoons in old combs maintain close contact with each worker for at least 20 d during development [29,30]. Therefore, the concentration of heavy metals in these cocoons also reflects the negative impact of old combs on bee health [60]. Specifically, the concentrations of Cr, Cd, Pb, Mn, Ni, and As in cocoons derived from old combs of *A. c. cerana* were significantly higher than those in newly built brood cells. Moreover, the heavy metal content and detoxification gene expression in 6-day-old larvae and newly emerged workers developing in these old combs were significantly increased.

The hydrophobic-lipophilic properties of comb constituents create differential environmental pollutant accumulation patterns between hive products. Due to their hydrophilic characteristics, certain contaminants preferentially partition into honey matrices [61]. Conversely, the lipophilic nature of beeswax and pollen facilitates the bioaccumulation of persistent organic pollutants, including miticides and veterinary pharmaceuticals used in apiculture [62]. This makes honeycomb a reliable bioindicator matrix for environmental monitoring, enabling simultaneous assessment of ecosystem contamination levels and apian population exposure risks [63]. For example, a study monitoring 11 heavy metals in bee products from colonies in the Campania region of Italy across three time periods (T1: COVID-19 pandemic lockdown; T2: restricted activity; T3: full reopening) found that the heavy metal content in bee products during the lockdown was significantly lower than that during the restricted activity and full reopening periods. This indicates that limiting industrial activities can help reduce pollutant levels both in the environmental and within bee colonies [64]. Similarly, the levels of environmental pollutants in colonies vary with human activity density, with higher Hg and Pb content found in honey from urban areas compared with that in mountainous regions [65]. Pollutants are ubiquitous in the environment, and when environmental pollutant levels are high, they are transferred into the hive, leading to increased pollutant levels in the combs, bee products, and bees themselves, which severely negatively impacts the quality of bee products and colony health [66,67]. Moreover, a critical concern is the widespread practice in the beekeeping industry of recycling beeswax from old combs into wax foundation sheets, which are reintroduced into hives [68]. These sheets often contain higher pollutant loads (like acaricides), and when provided to honeybee colonies, they may compromise colony health and survivorship [69].

## 4. Bee Adaptations in Old Combs

### 4.1. Behavioral Adaptations to Structural Degradation

**Figure 3:**
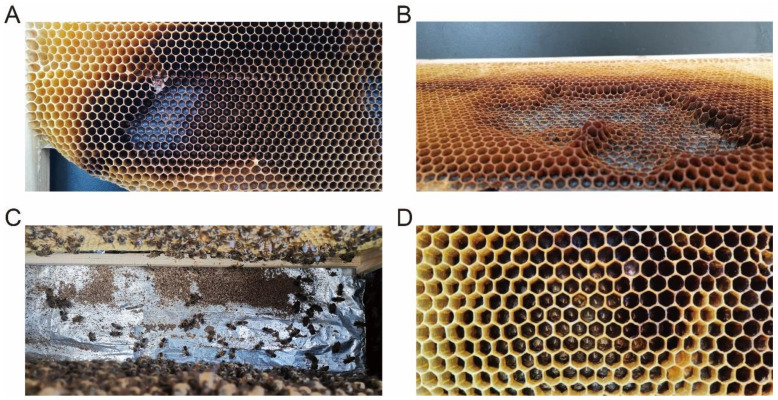
(**A**,**B**) Gnawed regions on old combs of *A. c. cerana*. (**C**) Wax residues accumulation at the hive bottom. (**D**) Comb cell reconstruction via wax secretion in gnawed areas.

Old combs impose significant negative impacts on individual bees, colony strength, and production performance. Thus, bee colonies may respond with different behaviors to mitigate the threats posed by old combs. For instance, in *A. mellifera* (housed in Moscow, Russia), the volume of brood cells decreases with successive generations of brood rearing. However, when the cell volume shrinks to 88% of that of new cells, workers meticulously clean the deposited residues within the cells and secrete wax to heighten the cell walls to reduce any further reduction and ensure minimum spatial requirements for normal brood development [70]. This cleaning and cell-wall extension strategy allows *A. mellifera* to counteract the developmental constraints caused by cell volume reduction in old combs. The Eastern honeybee (*A. cerana*) has evolved a distinct strategy, gnawing on old combs (Figure 3) [15]. Gnawing marks are frequently observed on the surface of old combs in *A. c. cerana* colonies, with abundant wax debris accumulating at the hive bottom. During periods of abundant nectar and pollen resources, workers rebuild the gnawed areas by secreting fresh wax, forming entirely new or semi-reconstructed cells. The dimensions of these semi-reconstructed cells and the morphological traits of the workers reared within them are significantly larger than those in old brood cells, demonstrating that gnawing and rebuilding old brood cells is an effective comb renewal strategy [11].

### 4.2. Molecular Mechanisms of Pollutant Detoxification

To combat environmental pollutants, bees have evolved detoxification enzyme gene families that metabolize both endogenous compounds and xenobiotics, including heavy metals, pesticides, and chemical carcinogens [71]. The primary detoxification enzyme superfamilies in bees include cytochrome P450 monooxygenases, carboxylesterases, and glutathione S-transferases [8]. Among these, the CYP450 gene family plays a crucial role in enhancing insect tolerance to environmental stressors and is recognized as a biomarker for pollutant exposure [65]. CYP450 monooxygenases exhibit peroxidase-like activity, protecting against reactive oxygen species (ROS) [72]. Glutathione S-transferases conjugate glutathione’s thiol group to ROS or electrophilic compounds, increasing their hydrophilicity and promoting cellular elimination [73]. The expression of these enzymes is tightly regulated by transcription factors and signaling pathways, including AhR/ARNT, CncC/Keap1, GPCR, and MAPK/CREB, which enhance detoxification capacity and stress tolerance [74].

## 5. Beekeeping Management Practices

Old combs negatively impact colony health and productivity. Therefore, to safeguard bee populations and promote efficient apiculture, targeted management practices are essential. Researchers recommend timely replacement of old combs to mitigate their negative impacts. In *A. mellifera*, removing combs older than 3 years facilitates the rearing of larger workers and supports optimal productivity [3,4]. In *A. cerana*, combs older than 6 months or those used for over eight brood generations should be replaced to promote individual development and colony growth [25]. Delayed comb replacement in *A. cerana* colonies can trigger comb-gnawing behavior, leading to wax debris accumulation. This debris creates an ideal environment for wax moths (*Galleria mellonella*) to lay eggs and fosters larval infestation [75]. Wax moth larvae burrow into old combs, excavating tunnels that result in pupal mortality [76]. Therefore, infestations can impair colony reproduction and may lead to absconding, causing substantial economic losses. Thus, beekeepers are advised to implement regular comb replacement protocols. When replacement is delayed, thorough hive cleaning is imperative to eliminate pest infestations and harmful microbial infections.

Importantly, the replacement of old combs necessitates proper disposal to mitigate potential contamination risks. These aged combs are typically processed for beeswax recovery, which is subsequently reintroduced into industries utilizing wax-based products, such as the production of foundation sheets to support colony development [68]. To ensure the safety and quality of recycled beeswax, appropriate physicochemical treatments are essential to reduce or eliminate contaminant levels. Recent studies have demonstrated that beeswax filtered using Norit SA4 PAH-activated carbon and Tonsil 114 FF diatomaceous earth achieves significant reductions in acaricides (e.g., chlorfenvinphos, coumaphos) and heavy metals (e.g., arsenic, mercury), while the hydrocarbon and monoester contents remain statistically unaltered [77]. However, it is noteworthy that total phenolic compounds, flavonoids, and the antioxidant capacity of lipophilic extracts exhibit slight reductions following treatment. Furthermore, an extraction process employing methanol at 65 °C, followed by an adequation process with water at 70 °C, has been shown to effectively reduce contamination levels of acaricides and pesticides in beeswax [78]. The decontaminated beeswax produced through this method exhibits quality parameters comparable to those of virgin beeswax. Notably, 80% of the methanol used in the extraction process is recyclable, and the resulting waste is manageable with minimal environmental impact. Despite these advancements, the development of advanced decontamination methodologies with superior comprehensive efficacy, minimized residual contamination, and enhanced processing efficiency remains a critical research priority. Further in-depth investigations are warranted to address these challenges and optimize the sustainability of beeswax recycling processes.

## 6. Conclusions

As the foundation of bee life, combs directly influence colony reproduction and productivity. Prolonged comb usage alters their physical and chemical properties, affecting individual development and overall colony health. Structural degradation of old combs results in changes to worker morphology and reduces foraging efficiency. Furthermore, pollutant accumulation in old combs negatively impacts bee health and compromises product quality. Despite these challenges, bees exhibit adaptive strategies. *Apis mellifera* maintains cell functionality through cleaning and wall extension, while *A. cerana* rebuild cells by gnawing on old combs. These behaviors underscore the resilience of honeybees and can inform apicultural management practices. Regular comb replacement reduces pollutant accumulation, improves developmental conditions, and enhances colony performance. With the decline in bee populations, identifying threats remains imperative. Future research should explore the application of advanced detection technologies, molecular biology, and multi-omics approaches to better understand pollutant profiles, their impacts on bee health, and bee resistance mechanisms. Such insights could guide strategies for improving bee health, such as identifying biomarkers or modulating gut microbiota. Furthermore, it is worth further exploring the efficient decontamination method of beeswax. In conclusion, maintaining high-quality combs is essential for colony vitality. Therefore, effective comb management not only boosts apicultural efficiency but also contributes to biodiversity conservation and ecological balance.

## Figures and Tables

**Table 1:** Diameter of brood cells of each bee species in the genus *Apis*.

Honeybee Species		Average Cell Diameter (mm)		Citation
		Worker	Drone	
*Apis mellifera*	*A. m. carnica*	5.51	6.91	Ruttner, 1988 [35]
	*A. m. ligustica*	5.49	6.62	Yang et al., 2021 [20]
	*A. m. scutellata*	4.80	6.15	Smith et al., 1961 [36]
	*A. m. jemenitica*	4.75	6.19	Dutton et al., 1981 [37]
	*A. m. litorea*	4.62	6.15	Ruttner, 1988 [35]
*Apis cerana*	*A. c. cerana*	4.82	5.62	Yang et al., 2021 [20]
	*A. c. japonica*	4.78	/	Ruttner, 1988 [35]
	*A. c. indica*	4.37	/	Ruttner, 1988 [35]
*Apis dorsata*		5.32	/	Khan et al., 2007 [38]
*Apis laboriosa*		6.3	/	Starr, 2021 [39]
*Apis florea*		2.7–3.1	4.2–4.8	Free, 1981 [40]
*Apis andreniformis*		2.8	4.2	Starr, 2021 [39]

## Data Availability

No new data were created or analyzed in this study. Data sharing is not applicable to this article.
